# Olfaction: An Overlooked Sensory Modality in Applied Ethology and Animal Welfare

**DOI:** 10.3389/fvets.2015.00069

**Published:** 2015-12-03

**Authors:** Birte L. Nielsen, Tadeusz Jezierski, J. Elizabeth Bolhuis, Luisa Amo, Frank Rosell, Marije Oostindjer, Janne W. Christensen, Dorothy McKeegan, Deborah L. Wells, Peter Hepper

**Affiliations:** ^1^INRA, UR1197 NeuroBiologie de l’Olfaction, Jouy-en-Josas, France; ^2^Institute of Genetics and Animal Breeding, Polish Academy of Science, Jastrzebiec, Poland; ^3^Adaptation Physiology Group, Wageningen University, Wageningen, Netherlands; ^4^Department of Evolutionary Ecology, Museo Nacional de Ciencias Naturales, CSIC, Madrid, Spain; ^5^Department of Environmental and Health Studies, Telemark University College, Bø, Norway; ^6^Department of Chemistry, Biotechnology and Food Science, Norwegian University of Life Sciences, Ås, Norway; ^7^Department of Animal Science, Aarhus University, Aarhus, Denmark; ^8^Institute of Biodiversity, Animal Health and Comparative Medicine, University of Glasgow, Glasgow, UK; ^9^School of Psychology, Queen’s University Belfast, Belfast, UK

**Keywords:** odors, chemoreception, behavior, feeding, stress, housing, reproduction, disease

It has long been known that odors and olfaction play a major role in behavioral development and expression in animals. The sense of smell is employed in numerous contexts, such as foraging, mate choice, and predation risk assessment. Indeed, olfaction is the primary sensory modality for most mammals, and many domestic species kept by humans, including chickens (1). Odors are therefore likely to influence many of the handling and management procedures carried out with animals, whether on farms, in zoos, in the laboratory, or in the family home. Despite this, applied ethologists and animal welfare scientists have not to any great extent investigated chemosensory perception or included odors in their studies.

One reason for this may be that olfaction is not considered a major sense in humans. However, odors are important for humans, too. Olfaction has a huge influence on the flavor of food (what we think of as taste is actually mostly olfaction). Memory and odors are very strongly linked, with certain odors evoking distant memories, such as when a musty odor is reminiscent of visits to your grandmother’s living room (2). Odors and olfaction may have much more influence on our own well-being than we think. For example, odors may serve to rekindle traumatic memories in individuals with PTSD (3). It has been estimated that 1 in 140 people suffers from anosmia, i.e., the total or partial loss of the sense of smell. Around 30% of these are thought to be clinically depressed (4), and anosmia is commonly used as a rat model for depression. Humans are also better at smelling than we think; humans are able to track a scent just like a dog if we get our noses close to the odor trail (5), and during sleep, we are able to learn to associate an odor with a sound (6). Given that human olfactory capacity is better than expected and very influential in our daily life – and this in a species that considers the sense of smell of little importance – how much *more* important are odors to the animals that we manage? Perhaps our (human) lack of recognition of the importance of olfaction in guiding our behavior and well-being, despite evidence to the contrary, has shaped our attitudes to the role that it might play in animals.

Another reason that olfaction has been studied so sparsely in non-laboratory species is because airborne chemical stimuli are inherently difficult to work with, measure, and control. As scientists, we have therefore focused on the human primary senses of vision and audition when testing responses to the environment in other species. It is easier to present an animal with the choice between a black and a white bucket, or to carry out tests in an arena using different sounds without risking that the animals drag residual sounds back into their home pen. However, given the importance of olfaction to most animals, odor stimuli may be more biologically relevant when performing behavioral tests. As odors form a major component of the animals’ surroundings, they cannot – and should not – be ignored whenever our aim is to improve animal welfare. Unfortunately, as well as the knowledge being scarce, the research is fragmented, and fundamental studies of olfaction are seldom carried out in collaboration with colleagues working on more applied aspects of olfactory behavior (7).

At least five areas concerning the handling and management of domestic and captive animals exist where odors are likely to play an important role (Figure 1). It is no surprise that both *feeding and foraging* are heavily influenced by smells. The perception and processing of chemosensory information related to food starts already before birth or hatching (8, 9). For example, feeding sows a flavored food during late gestation reduces stress and improves the health and growth of their piglets if these are exposed to the same odor post-weaning (10, 11). Further study is needed on the use of odorants to encourage foraging and to stimulate food intake, particularly post-weaning, as well as on the link between odors and neophobia.

**Figure 1:**
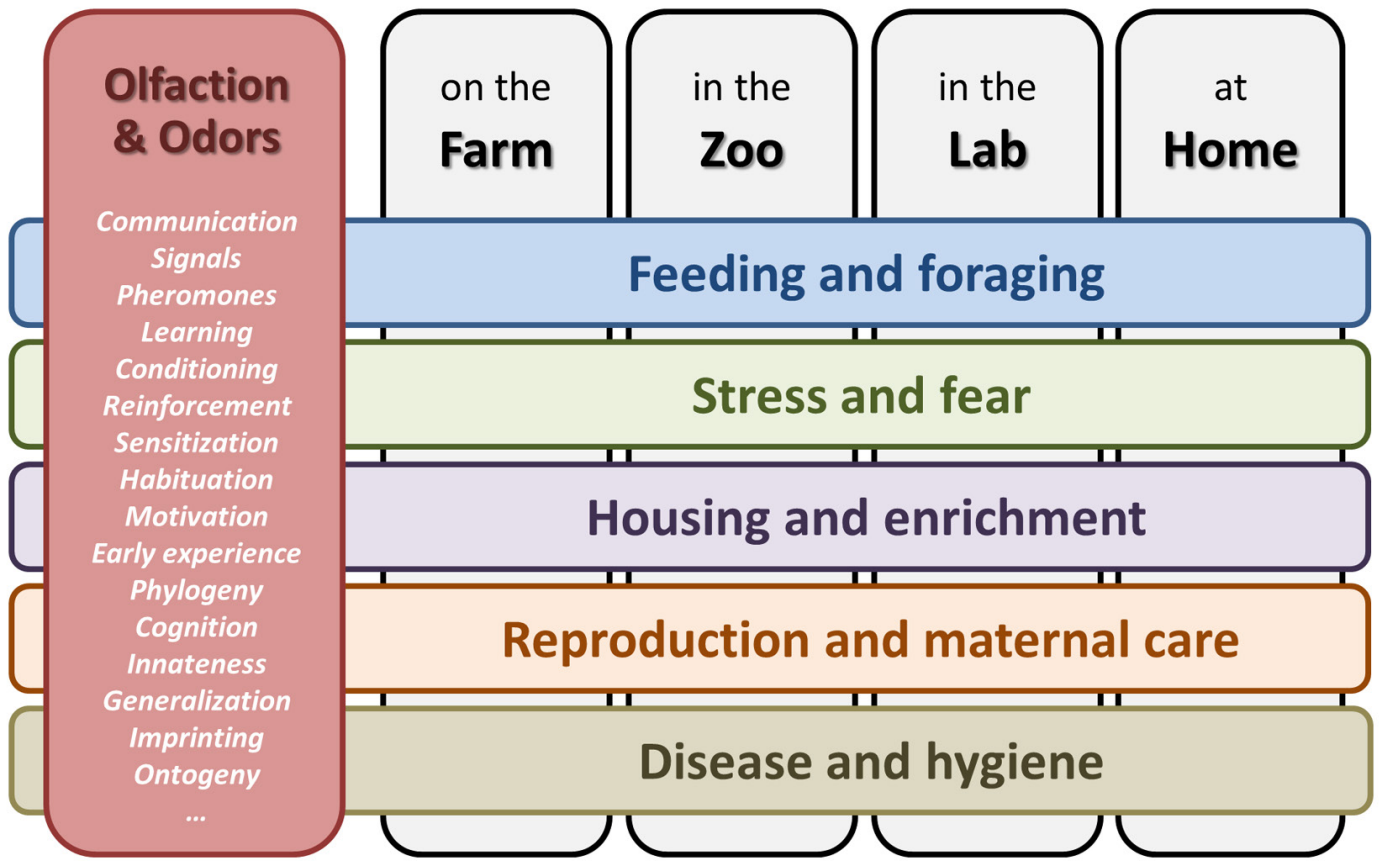
**Five areas (horizontal bars), where olfaction and odors influence the behavior and welfare of animals, kept in different human environments (vertical gray bars)**. Behavioral and olfactory concepts involved are listed in the red box on the left.

*Stress and fear* is another area of interest, as odorants could potentially be used to reduce negative affective states that may arise with animal handling. The identification of stress-inducing odors and those emitted during stressful situations are important areas where our current knowledge is very limited. There are indications that pigs are sensitive to the emotional state of pen mates in the absence of visual information (12); if odors from animals in distress have an impact on conspecifics, then negative states could spread beyond the animal enclosure in zoos and labs, and on farm.

Odors are ideal as a form of *environmental enrichment* as they take up no room and are relatively cheap. They can be made dynamic both in time and space, thereby creating the novelty aspect that is missing in many enrichment attempts. In this manner, we could create species-specific odor-scapes for captive animals, which are composed of different and fleeting smells similar to those experienced as they traverse their natural environment including both positive and negative odors (e.g., predators they would naturally experience and avoid). In this regard, Wells (13) encourages the use of harmless, non-stressful stimuli that target the dominant sense of the species concerned, which in many cases is olfaction. Clark and King (14) encourage more empirical quantification of the use of olfactory stimulation in zoo animals; odors have been found to increase behavioral diversity and activity levels in captive black-footed cats, but odors have little effect on the behavior of captive gorillas (15, 16).

In terms of *reproduction*, studies have focused mainly on the use of odorants to stimulate libido [e.g., cattle (17)], identification of odors associated with reproductive states [e.g., rats (18, 19)], and olfactory aspects of *maternal care* [e.g., offspring acceptance, fostering, and suckling; (20)]. An example of this is the suckling response of newborn mice, which is elicited by signature odors in the amniotic fluid that are learned and recognized prior to first suckling (21). Finally, many aspects of *disease and hygiene* are potentially associated with smells, such as the role of odors as auxiliary diagnostic symptoms of certain animal diseases (22); and as attractants/repellents for insects, which are both disease vectors and sources of irritation. Indeed, the presence of some smells may induce “noise” in the chemosensory environment, potentially interfering with olfactory perception and communication.

So, how can the study and use of odors in applied ethology and animal welfare science be advanced? First, all studies should bear in mind the potential impact of odors. The invisible influence of odors, undetected by the human experimenter may explain unexpected results. Second, if odors are to be used to improve the way we manage animals, then the current knowledge on olfaction in a variety of species must be synthesized using a common template and language. This would require participation from across scientific disciplines with expertise in areas associated with olfaction, including applied ethologists, neuroscientists, conservation biologists, and chemists. However, different scientific disciplines are separated in terms of scientific aims, methodologies, and the technical language used. Thus, a third action will be the provision of multidisciplinary forums, such as conferences and blogs where researchers from various disciplines can share and compare knowledge, techniques, and practices. Animal welfare research would benefit from adopting as well as *adapting* methods developed by neuroscientists for testing olfactory capacity and preferences. Through such collaborations, neuroscientists would also get an insight into the application of behavioral science outside of the rodent cage. This shared knowledge base can then be used to plan studies that better understand the role, and use, of odors in applied ethology and animal welfare.

In summary, to improve animal welfare as well as reproduction in domestic and captive animals, olfaction and odors should be taken into account to a much larger extent than is presently the case. By using the right (or removing the wrong) odors at the right time in the housing and handling of farm, zoo, lab, and companion species, we may be able to improve various aspects of animal behavior, reproduction, and health, and create animal environments that are more suitable, more productive, as well as welfare enhancing.

## Author Contributions

BN initiated the discussion of the subject and the writing of this paper. TJ, JB, LA, FR, MO, JC, DM, DW, and PH contributed significantly to the discussion of the subject, and the development, writing, and final version of this paper.

## Author Note

This article is based on a (technically disastrous) oral presentation given by the first author at the 48th Congress of the International Society for Applied Ethology (ISAE) in 2014; in turn inspired by an (unsuccessful) COST action application jointly submitted by the authors in 2012 and 2013. We do not give up easily.

## Conflict of Interest Statement

The authors declare that the research was conducted in the absence of any commercial or financial relationships that could be construed as a potential conflict of interest.
